# Targeted RNAi screen reveals novel regulators of RNA-binding protein phase transitions in *Caenorhabditis elegans* oocytes

**DOI:** 10.1093/g3journal/jkaf266

**Published:** 2025-11-10

**Authors:** Mohamed T Elaswad, Grace M Thomas, Corrin Hays, Nicholas J Trombley, Jennifer A Schisa

**Affiliations:** Biochemistry, Cell and Molecular Biology Program, Central Michigan University, Mount Pleasant, MI 48859, United States; Department of Biology, Central Michigan University, 1455 Calumet Court, Mount Pleasant, MI 48859, United States; Department of Biology, Central Michigan University, 1455 Calumet Court, Mount Pleasant, MI 48859, United States; Department of Biology, Central Michigan University, 1455 Calumet Court, Mount Pleasant, MI 48859, United States; Department of Biology, Central Michigan University, 1455 Calumet Court, Mount Pleasant, MI 48859, United States; Biochemistry, Cell and Molecular Biology Program, Central Michigan University, Mount Pleasant, MI 48859, United States; Department of Biology, Central Michigan University, 1455 Calumet Court, Mount Pleasant, MI 48859, United States

**Keywords:** *Caenorhabditis elegans*, oocyte quality, phase transitions, MEX-3, RNA-binding proteins, condensates, Animalia, WormBase

## Abstract

The ability of oocytes to maintain their quality is essential for successful reproduction. One critical aspect of oocyte quality and successful embryogenesis after fertilization is the proper regulation of the stores of maternal mRNA by RNA-binding proteins. Many RNA-binding proteins undergo regulated phase transitions during oogenesis, and alterations of the protein phase can disrupt its ability to regulate mRNA stability and translation. In *Caenorhabditis elegans*, regulators of RNA-binding protein phase transitions in maturing oocytes of young adult hermaphrodites remain poorly characterized. However, a few recently identified genes are also required for the clearance of damaged proteins during maturation, suggesting coordination between these processes. To explore this relationship and gain insight into the regulation of phase transitions, we conducted a targeted RNAi screen of genes required for removal of protein aggregates in maturing oocytes. Here, we identify 6 novel regulators of phase transitions of the KH-domain protein MEX-3. We present strong evidence that the regulation of MEX-3 phase transitions in the oocyte overlaps with, but is distinct from, the regulatory network of protein aggregate clearance.

## Introduction

The regulation of maternal mRNAs by RNA-binding proteins is essential for oocyte growth and early embryogenesis across metazoa (reviewed in Conti and Kunitomi 2024). In the absence of tight spatial and temporal regulation of mRNA stability and translation, birth defects and infertility can arise. Increasing evidence indicates that mRNA regulation depends on the organization of RNA and RNA-binding proteins into membraneless organelles formed by phase separation (Holehouse and Alberti 2025). During oogenesis, many RNA-binding proteins and maternal mRNAs undergo dynamic phase transitions, alternating between decondensed and condensed states as oocytes progress through meiosis and respond to environmental or physiological cues (Schisa et al. 2001; Jud et al. 2008; Flemr et al. 2010; Cheng et al. 2022). The regulation of phase transitions is critical for function. For example, perturbing the solid phase of *oskar* RNP granules in *Drosophila* oocytes disrupts *oskar* mRNA localization and translation, leading to defective embryos (Bose et al. 2022). In mammals, disruption of mitochondria-associated ribonucleoprotein domain (MARDO) condensates in GV-stage oocytes causes premature maternal mRNA degradation (Cheng et al. 2022). Two MARDO components, DDX6 and LSM14, undergo phase transitions in *Caenorhabditis elegans* and other species, which suggests evolutionary conservation of the processes regulating maternal mRNAs (Ladomery et al. 1997; Nakamura et al. 2001; Navarro et al. 2001; Audhya et al. 2005; Boag et al. 2005; Squirrell et al. 2006; Jud et al. 2008; Noble et al. 2008; Hubstenberger et al. 2013; Elaswad, Watkins, et al. 2022).

**Fig. 1.**
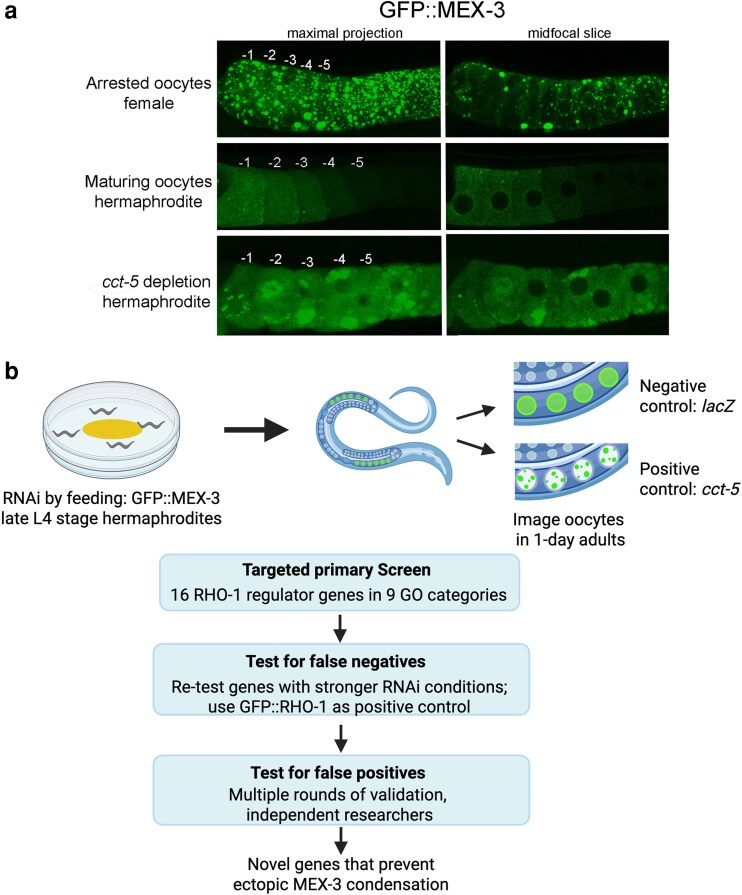
Rationale and workflow for targeted RNAi screen to identify regulators of MEX-3 phase transitions in oocytes. a) The KH-domain protein MEX-3 is dynamic; while it is mostly decondensed in the maturing oocytes of young hermaphrodites, MEX-3 undergoes dramatic condensation into large granules of oocytes undergoing an extended meiotic arrest, e.g. in females lacking sperm. The most proximal *C. elegans* oocyte, closest to the sperm, is referred to as the −1 oocyte. The extent of arrest can be seen when comparing the positions of the 5 most proximal, maturing, and arrested oocytes. A maximal confocal projection and single z-slice are shown for comparison. Scale bar is 10 µm. b) The targeted RNAi screen used a GFP::MEX-3 strain (DG4269), where L4-stage hermaphrodites were moved onto plates with RNAi bacteria. After 24 h, oocytes in 1-d adults were imaged using confocal microscopy. The negative control was *lacZ*, and the positive control was *cct-5.* In the primary screen, 16 RHO-1 regulator genes were depleted, one at a time. To test for false negatives, negative genes were screened using stronger RNAi conditions, and depletions were performed in parallel in GFP::RHO-1 to ensure efficacy of gene expression depletion. Positive hits were re-screened in at least 3 rounds of validation by multiple researchers. The screen uncovered novel genes that prevent ectopic condensation of MEX-3. Created in BioRender. Schisa (2025)  https://BioRender.com/an863wa.

The *C. elegans* model offers many advantages in studying post-transcriptional gene regulation including the ability to conduct functional RNAi screens to identify regulators of phase transitions in oocytes (Hubstenberger et al. 2015; Wood et al. 2016). Studying the KH-domain protein MEX-3 is of high interest due to its dynamic phase transitions during development and key role as a translational repressor in early embryos. MEX-3 is relatively decondensed in maturing oocytes of young hermaphrodites, but it reversibly condenses into large condensates when oocytes halt meiotic maturation, e.g. when hermaphrodites become depleted of sperm during development (Fig. 1; Schisa et al. 2001; Jud et al. 2008). Understanding the regulation of MEX-3 phase transitions is also of high interest because in colorectal cancer cells unregulated hMex3 phase transitions lead to mRNA degradation and poor patient outcomes (Chen et al. 2024).

A decondensed MEX-3 phase correlates with activated Extracellular signal-Regulated Kinase (ERK), Major Sperm Protein signals from sperm, and dispersed ER architecture in maturing oocytes (Jud et al. 2008; Patterson et al. 2011). The maintenance of decondensed MEX-3 and dispersed ER in oocytes requires the RNA helicase CGH-1/DDX6, the CCT chaperonin, and actin (Langerak et al. 2019; Elaswad et al. 2024). For example, depletion of the CCT chaperonin subunit *cct-5* by RNAi results in ectopic MEX-3 condensates and precocious translation of maternal mRNA in maturing oocytes (Fig. 1, Elaswad et al. 2024). Interestingly, similar correlations between sperm signals and activated ERK are observed with the clearance of protein aggregates in maturing oocytes. RHO-1 protein aggregates form if oocytes halt meiotic maturation, and their clearance in maturing oocytes requires CGH-1, the CCT chaperonin, and actin (Bohnert and Kenyon 2017; Samaddar et al. 2021). The shared characteristics between regulators of MEX-3 phase transitions and aggregate clearance suggest a possible shared network controlling oocyte maturation processes.

To test this hypothesis, we screened genes previously implicated in the clearance of protein aggregates for roles in MEX-3 regulation (Samaddar et al. 2021). Our RNAi-based analysis focused on 16 genes across 9 gene ontology categories. Here, we identify *copb-2*, *sar-1*, *sec-24.1*, *let-711*, *pbs-7*, and *rpn-6.1* as novel regulators that prevent ectopic condensation of MEX-3 in maturing oocytes. In contrast, genes involved in lysosome acidification, regulation of mitochondrial membrane potential, calcium ion transport, and ESCRT complex-mediated autophagy do not appear to be required to modulate MEX-3 phase transitions. Taken together, our results demonstrate that while the regulatory networks maintaining MEX-3 phase and protein aggregate clearance in maturing oocytes intersect, they are distinct.

## Methods

### Worm strains and maintenance

*Caenorhabditis elegans* strains were maintained on Nematode Growth Medium (NGM) plates and fed OP50  *Escherichia coli* at 20 °C, unless specified (Brenner 1974). The following strains were used: DG4269 (*tn1753[gfp::3xflag::mex3]*) and SA115 (*unc-119(ed3) III; tjIs1[pie-1::GFP::rho-1 + unc-119(+)]*). Strains were synchronized before experiments using a hypochlorite bleaching method (Stiernagle 2006). WormBase was used to assist in planning experiments Sternberg et al. (2024).

### RNA-mediated interference screen design

Feeding RNAi was performed as described previously (Timmons and Fire 1998). Transformed RNAi clones of HT115  *Escherichia coli* expressing dsRNA for the target gene from the Source Bioscience RNAi library (Kamath and Ahringer 2003) were used to deplete gene expression by feeding. Each colony of bacteria was grown in LB with 50 μg/mL carbenicillin at 37 °C for 7 h. Plates with RNAi media (NGM containing 50 μg/mL carbenicillin and 1 mM IPTG [isopropyl β-D-1-thiogalactopyranoside]) were seeded with the liquid culture. dsRNA expression was induced at room temperature overnight, and the plates were blinded. All RNAi clones were verified by sequencing.

In the primary screen, we tested 16 genes in 9 of the GO categories identified as RHO-1 regulators (Fig. 1, Table 1). Our standard RNAi conditions in the primary screen did not include any supplemental IPTG, which is sometimes added to liquid RNAi bacterial subcultures to induce higher levels of dsRNA. Late L4 (fourth larval)—stage GFP::MEX-3 hermaphrodites were placed on RNAi media at 24 °C for 24 h before collecting confocal images of oocytes in 1-d post-L4 adults. The negative control was *lacZ(RNAi)*, where MEX-3 is largely diffuse in oocytes. The positive control was *cct-5(RNAi)*, where MEX-3 ectopically condenses (Elaswad et al. 2024). Candidates for positive hits were defined as gene depletions yielding ectopically condensed MEX-3 in oocytes of at least half of the five 1-d post-L4 hermaphrodites scored. We noted the phenotypes of ectopic MEX-3 condensates included small, spherical granules with or without accompanying strong enrichment of MEX-3 at the oocyte cortex.

**Table 1.** RNAi screen identifies novel regulators of MEX-3 phase transitions and distinguishes MEX-3 regulation from the network regulating clearance of RHO-1 protein aggregates.

Biological process	RNAi gene target	Primary screen (condensed MEX-3)	Quantitation after validation^a^	Significant change	Regulates RHO-1 (our study)
Protein degradation	*pbs-7*	Yes	100%C	+	ND
	*rpn-6.1*	Yes	57%C	+	ND
Protein synthesis	*rpl-3*	No	45%C	−	ND
RNA processing	*let-711*	Yes	100%G	+	ND
	*ess-2*	No	ND	ND	ND
Vesicle-mediated trafficking	*copb-2*	Yes	100%G	+	ND
	*sec-24.1*	Yes	92%G	+	ND
	*sar-1*	Yes	90%G	+	ND
V-ATPase subunits	*vha-12*	No	21%G	−	Yes
	*vha-2*	No	20%G	ND	Yes
ATP synthase	*atp-3*	No	0%	−	Yes
Ca^2+^ ion transport	*sca-1*	No	0%	−	Yes
	*itr-1*	No	ND	ND	ND
ESCRT subunits	*vps-37*	No	0%	−	Yes
	*vps-54*	No	ND	ND	ND
ER	*spcs-1*	No	ND	ND	No

^a^Quantitation was performed after validation, i.e. at least 3 replicates were performed by independent researchers. The percent of worms with condensation > control is indicated. C following the percent is a cortical phenotype where MEX-3 was enriched strongly at the oocyte cortex and in a small number of granules. G indicates the major phenotype was ectopic condensation of MEX-3 into spherical granules throughout the cytoplasm. ND is not determined.

To minimize false negatives, we re-tested the negative genes using stronger RNAi conditions where liquid bacterial cultures were supplemented with 5 mM IPTG for 45 to 60 min prior to plating on RNAi media that also included the standard 1 mM IPTG. As a readout of the RNAi efficacy in depleting expression of the intended target genes, we tested each depletion with GFP::RHO-1 worms in parallel to testing GFP::MEX-3 worms. Depletions of 5 genes of 6 tested resulted in the expected phenotype of ectopic RHO-1 aggregates (Samaddar et al. 2021). For one gene, *spcs-1*, we did not reproduce the ectopic RHO-1 phenotype across 3 replicates. To test for false positives and validate candidate positive hits, at least 2 independent researchers tested positive hits in at least 3 replicates.

### Microscopy and image analysis

Confocal Z-stacks of oocytes were collected on a Nikon A1R confocal microscope system. Worms were picked onto slides made with 2% agarose pads and paralyzed using 6.25 mM levamisole. All images were collected using identical levels and settings. Worms on each slide were imaged within 10 min of mounting on slides to avoid inadvertent stress responses (Elaswad, Munderloh, et al. 2022).

### Phenotype scoring

After the validation step, we used both qualitative and quantitative methods to score MEX-3 phenotypes. The percent of worms with increased condensation of MEX-3 in oocytes, compared to the control, is indicated (Table 1). A “C” following the percent is a cortical phenotype where MEX-3 was enriched strongly at the oocyte cortex and in a small number of granules. “G” indicates the major phenotype was ectopic condensation of MEX-3 into spherical granules throughout the cytoplasm. To determine if the distribution of MEX-3 became significantly more condensed than in the negative control, we used the skewness analysis tool in Fiji (ImageJ). After extracting mid-focal and cortical z-slices from z-stacks, we drew an ROI around each of the 3 most proximal oocytes, excluding the nucleus. Skewness is a measure of the uniformity of fluorescence signal in an ROI. A value of zero indicates complete symmetry of fluorescent pixels within the cell, i.e. no significant condensation. Skewness values deviating from zero indicate increasingly asymmetric distributions of fluorescent pixels within a cell, i.e. a proxy for condensation (Elaswad et al. 2024). The mean skewness value for 3 oocytes was calculated for each worm. Statistical analyses are described below. Significant MEX-3 condensation was detected for 6 of 7 depletions. In depletions with ectopic condensates, we used Fiji to calculate the integrated density divided by the area of the ROI as a measure of the mean fluorescence intensity of MEX-3 in the −1 to −3 oocytes.

### Statistical analysis

Sample sizes were determined using G*Power 3.1 for power analyses; all experiments were blinded and done at least in triplicate. Data are presented as mean ± SEM unless otherwise indicated. Statistical analyses were performed on GraphPad Prism 10.5, and specific tests are noted in figure legends. The Dunn's correction was used with Kruskal–Wallis tests to control the type I error rate. Corrected *P*-values are presented, and *P*-values <0.05 were considered statistically significant.

## Results and discussion

### Design of targeted RNAi screen to identify regulators of MEX-3 phase transitions in maturing oocytes

To determine the extent to which the genes required to prevent RHO-1 aggregates in maturing oocytes also prevent ectopic condensation of RNA-binding proteins, we performed a targeted RNAi screen. We selected 16 candidate genes across 9 Gene Ontology categories/biological processes implicated in preventing protein aggregates during oocyte maturation (Samaddar et al. 2021) (Table 1). We investigated the KH-domain RNA-binding protein MEX-3 because it is evolutionarily conserved, with 4 mammalian homologs, and exhibits dynamic phase transitions in vitro and across species in vivo (Donnini et al. 2004; Buchet-Poyau et al. 2007; Jud et al. 2008; Wood et al. 2016; Elaswad et al. 2024). MEX-3 is highly condensed into granules in the stacked, meiotically arrested oocytes of aged hermaphrodites depleted of sperm or females (Fig. 1a) (Jud et al. 2007). If a female mates with a male, meiotic maturation resumes, and MEX-3 decondenses (Jud et al. 2008). In contrast, the MEX-3 protein is largely decondensed throughout the cytosol of the maturing oocytes of young hermaphrodites (Draper et al. 1996; Fig. 1a). Note, the most proximal *C. elegans* oocyte, which is positioned for meiotic maturation, ovulation, and fertilization, is referred to as the −1 oocyte.

In our screen, the negative control was *lacZ(RNAi)*, and the positive control was *cct-5(RNAi)* in which patchy MEX-3 condensates accumulate near cortical membranes, and increased numbers of small spherical granules appear. The *cct-5(RNAi)* oocytes do not appear to be in extended meiotic arrest based on the unstacked oocyte morphology (see details in Elaswad et al. 2024; Fig. 1a). A positive hit was broadly defined in the primary screen as ectopic condensation of MEX-3 in the maturing oocytes of at least 50% of worms. The primary screen was followed by tests for false negatives via replicates with stronger RNAi conditions and by testing effects of gene depletion on GFP::RHO-1 as a positive control in parallel. Testing for false positives included multiple rounds of validation of phenotypes by at least 2 independent researchers (Fig. 1b).

**Fig. 2.**
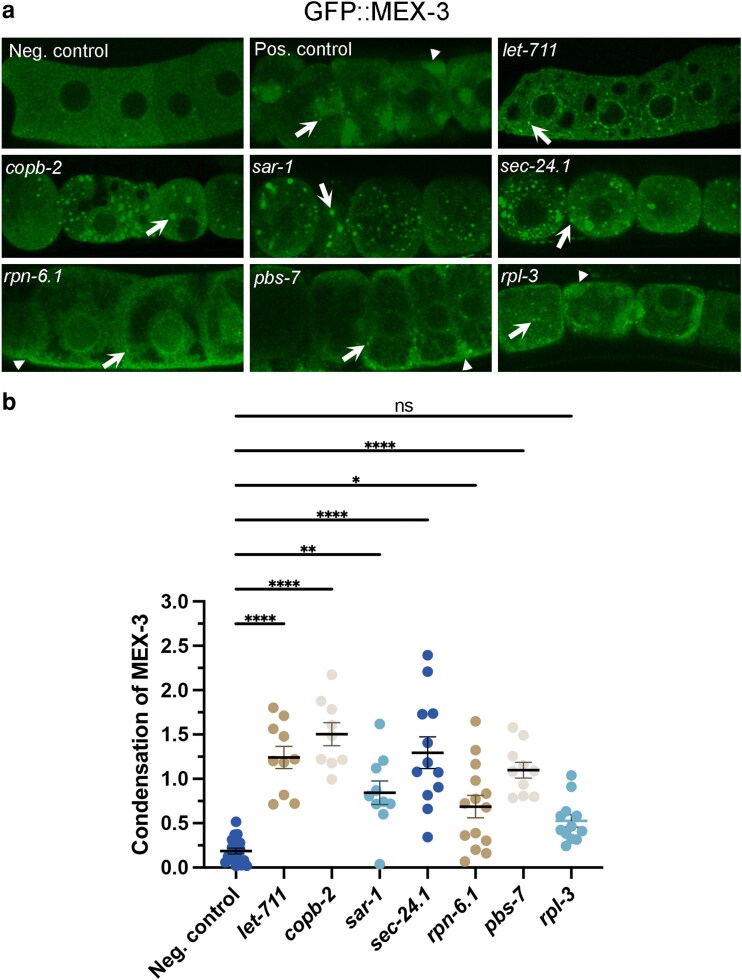
A subset of RHO-1 regulators prevents ectopic MEX-3 condensation in oocytes. a) Confocal images of GFP::MEX-3 in oocytes after RNAi depletions. MEX-3 is decondensed in the *lacZ(RNAi)* negative control. In the *cct-5(RNAi)* positive control, MEX-3 is condensed into small, spherical granules (arrows) and into larger heterogeneous condensates enriched at the cell periphery (arrowheads). Depletions of *let-711*, *copb-2*, *sar-1*, and *sec-24.1* resulted in ectopic spherical MEX-3 granules. Depletions of *rpn-6.1*, *pbs-7*, and *rpl-3* resulted in ectopic enrichment at the cell periphery and small numbers of granules. All micrographs are single-Z plane confocal images, and the most proximal oocyte is oriented to the left. Scale bar is 10 µm. b) Significant increases in MEX-3 condensation were detected after all depletions except *rpl-3* using the Fiji Skewness tool. Each dot on the graph represents the average of the 3 skewness values for the −1 to −3 oocytes in each worm. Higher skewness values indicate less uniformity of GFP signal in an ROI, which is a measure of increased condensation. Similar results were seen in both mid-focal and cortical Z slices (mid-focal values not shown). Kruskal–Wallis test. * *P* < 0.05, ** *P* < 0.01, **** *P* < 0.0001. *n* = 10 to 15.

In the primary screen, we initially identified 6 positive candidates among the 16 genes: *let-711*, *copb-2*, *sar-1*, *sec-24.1*, *rpn-6.1*, and *pbs-7* (Table 1, Fig. 2a). We detected one additional candidate gene, *rpl-3*, after further testing for false negatives with more stringent RNAi conditions. All 7 genes were validated as positive candidates during multiple experiments performed by independent researchers. We qualitatively noted 2 categories of MEX-3 phenotypes: (i) increased numbers of ectopic spherical granules and (ii) increased enrichment at the cell periphery with a small number of ectopic granules (Table 1). We used the skewness tool in ImageJ as a measure of MEX-3 condensation (described in methods). Our quantitative analysis revealed statistically significant increases in MEX-3 condensation after depletion of 6 of the 7 genes (Fig. 2b).

### Novel regulators of MEX-3 phase transitions

#### Genes associated with protein degradation and protein synthesis

Depletion of 3 genes broadly involved in protein degradation and synthesis, *pbs-7*, *rpn-6.1*, and *rpl-3*, resulted in increased cortical enrichment of MEX-3 and produced small, ectopic spherical granules in the cytoplasm (Fig. 2). In these depletions, MEX-3 appeared to be excluded from regions of the cytoplasm, which could contribute to MEX-3 enrichment at the cell periphery. For 2 of these 3 gene depletions, altered ER architecture is induced (Samaddar et al. 2021), suggesting that MEX-3 may be excluded from regions of ectopic ER sheets in the cytoplasm. This possibility is bolstered by the observation that expanded ER sheets in the cytoplasm exclude RHO-1 in arrested oocytes (Langerak et al. 2019; Samaddar et al. 2021). While the condensation of MEX-3 was not statistically increased after *rpl-3(RNAi)*, nearly half of the worms displayed elevated MEX-3 condensation. *rpl-3* encodes the ribosomal protein L3, and similar ribosome genes promote the size of PGL-1 germ granules in embryos (Updike and Strome 2009). Interestingly, this role contrasts with our observations of *rpl-3* inhibiting condensation of MEX-3 in oocytes. In different developmental contexts, active translation may differentially modulate condensation or decondensation of RNA-binding proteins.

RPN-6.1 (ortholog of PSMD11) is a proteasome 26S subunit involved in ubiquitin-dependent degradation, and PBS-7 (ortholog of PSMB4) is part of the proteasome core complex. Ubiquitin binding can modulate phase transitions of UBQLN2 in vitro, and 21 proteasomal and ubiquitin genes promote condensation of PGL-1 in embryos (Updike and Strome 2009; Dao et al. 2018). In contrast, our results suggest the degradation machinery may also be needed to maintain a decondensed phase of MEX-3 in oocytes. Since protein accumulations beyond the concentration threshold required for phase separation can lead to ectopic condensation, we calculated mean MEX-3 fluorescence intensities in oocytes after depletion of all positive hits (Hyman et al. 2014; Alberti et al. 2017). Interestingly, none of the 7 gene depletions increased levels of MEX-3 (Supplementary Fig. 1), suggesting MEX-3 condensates may not be a simple consequence of overaccumulation.

#### Genes associated with RNA processing

The RNA helicase CGH-1/DDX6 has been shown to prevent both ectopic MEX-3 condensates and ectopic RHO-1 aggregates in maturing oocytes (Langerak et al. 2019; Samaddar et al. 2021). In our primary screen, we tested 2 additional RHO-1 regulators that function broadly in RNA processing. We did not detect changes in MEX-3 after depletion of *ess-2*; however, we detected increased ectopic MEX-3 condensates after depletion of *let-711/Not1*, an essential scaffolding protein in the CCR4-NOT deadenylase complex (Fig. 2). A role for LET-711/NOT1 in preventing ectopic MEX-3 condensation in maturing oocytes is similar to its role modulating the phase transitions of 2 other RNA-binding proteins in *C. elegans*. LET-711 prevents ectopic square, solid-like sheets of CAR-1/Lsm14 in arrested oocytes and inhibits the condensation of PGL-1 germ granules in maturing oocytes (Updike and Strome 2009; Hubstenberger et al. 2015). This general function may be conserved across species as Not1p promotes disassembly of P-bodies in yeast (Sachdev et al. 2019). Disrupted deadenylation in oocytes may lead to an accumulation of undegraded maternal mRNA which may in turn, nucleate ectopic condensates of RNA-binding proteins.

#### Genes associated with vesicle-mediated trafficking

We identified 3 genes involved in vesicle-mediated trafficking between the ER and Golgi as novel inhibitors of ectopic MEX-3 condensation. Depletion of *copb-2*, *sar-1*, and *sec-24.1* resulted in numerous spherical MEX-3 granules in unusually rounded oocytes (Fig. 2). COPB-2/β’Cop encodes a COPI-coat complex subunit that functions to transport proteins within Golgi membranes and from the Golgi to the ER (Beck et al. 2009). While no reports to our knowledge have identified proteins in the COPI complex as modulators of RNA-binding protein phase transitions in oocytes, 2 COPI proteins, Sec27p/β’Cop and Sec 21p/γCop, limit P-body formation in yeast (Kilchert et al. 2010). In considering possible mechanisms by which COPB-2 acts to modulate MEX-3 phase, it is intriguing to consider the range of environmental stresses that induce MEX-3 condensation (Jud et al. 2008; Elaswad, Watkins, et al. 2022). Since COPI complexes move improperly folded proteins from the Golgi to the ER, deletion of *copb-2* in oocytes may induce the Unfolded Protein Response or ER cellular stress response (Jud et al. 2008; Elaswad, Watkins, et al. 2022). Alternatively, studies suggest osmotic stress mediates the increase in P-bodies in yeast Sec mutants (Kilchert et al. 2010). Future studies are also needed to determine if COPB-2 modulates MEX-3 phase transitions independently or as part of the activity of COPI vesicles.

SAR-1/Sar1 is an ER-exit-site, small GTPase that initiates COPII vesicle formation and enables transport of cargo from the ER to Golgi as part of the secretory pathway (Barlowe and Schekman 1993; Barlowe et al. 1993). SAR-1 recruits either SEC-24.1/Sec24D or SEC-24.2 with SEC-23 to form the inner coat of the COPII vesicle and recruit cargo into COPII vesicles (Matsuoka et al. 1998; Bi et al. 2002; Sato and Nakano 2007). Four COPII complex proteins were recently shown to regulate the organization of P-bodies in *Drosophila* nurse cells, the cells that support oocyte growth (Milano et al. 2025). Depletions of Sec23, Sec13, or Sec31 result in increased condensation of 2 P-body proteins; however, depletion of Sar1 results in reduced condensation of P-body proteins. Notably, our finding that SAR-1 prevents condensation of MEX-3 in *C. elegans* oocytes contrasts with the *Drosophila* result, where it promotes condensation. However, it is similar to yeast studies where Sar1p limits the number of P-bodies in the cell (Kilchert et al. 2010). These differences may reflect cell-type-specific regulation or COPII-independent functions of SAR-1.

### Regulatory network of RNA-binding protein phase transitions differs from protein aggregates

Several RHO-1 regulators showed no detectable effect on MEX-3 phase. These included genes involved in lysosome acidification (*vha-2* and *vha-12*), an ATP synthase in mitochondria that is required to decrease the membrane potential (*atp-3*), calcium ion transport genes (*sca-1*, *itr-1*), genes in the ESCRT complex (*vps-37*, *vps-54*), and an ER homeostasis gene (*spcs-1*). Interpretations of negative results with RNAi must be undertaken cautiously as the efficacy of gene depletions by RNAi varies, and false negatives are possible (Timmons et al. 2001). To test for false negatives, we selected 1 or 2 genes in each of the 5 GO categories with negatives and performed RNAi in the GFP::MEX-3 and GFP::RHO-1 strains in parallel. We confirmed that each depletion induced the expected RHO-1 aggregation phenotype before cautious interpretation of negative MEX-3 results (Samaddar et al. 2021).

**Fig. 3.**
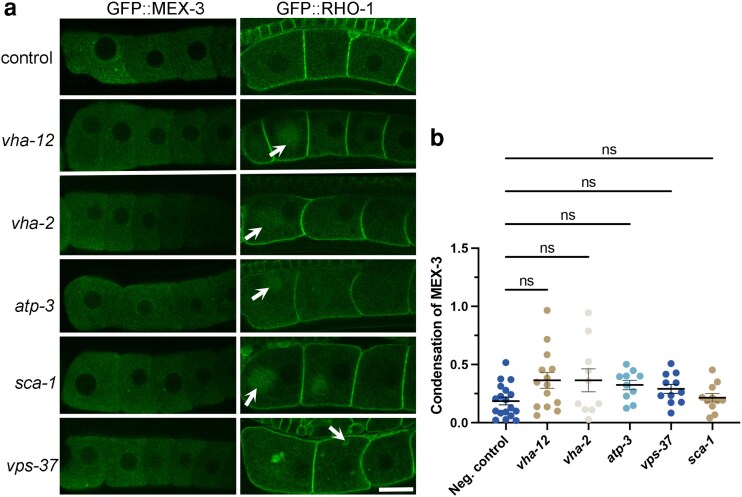
A subset of RHO-1 regulators is not required to prevent ectopic MEX-3 condensation in oocytes. a) *Left column)* GFP::MEX-3 appears mostly decondensed in *lacZ(RNAi)* negative control oocytes and after depletion of 5 RHO-1 regulator genes. *Right column)* The efficacy of each RNAi experiment was validated by parallel depletions in the GFP::RHO-1 strain. RHO-1 is enriched at the oocyte cortex of *lacZ(RNAi)* control oocytes. After depletion of the 5 genes, ectopic RHO-1 aggregates were detected (arrows). All micrographs are single-Z plane confocal images, and the most proximal oocyte is oriented to the left. Scale bar is 10 µm. b) No significant increases in MEX-3 condensation were detected after RNAi depletions using the FIJI Skewness tool. Each dot on the graph represents the average of the 3 skewness values for the −1 to −3 oocytes in each worm. Higher skewness values indicate less uniformity of GFP signal in an ROI, which is a measure of increased condensation. Similar results were seen in both mid-focal and cortical Z slices (mid-focal values not shown). Kruskal–Wallis test. ns is not significant. *n* = 10 to 15 worms.

In worms treated with RNAi of *vha-12*, *vha-2*, *atp-3*, *sca-1*, or *vps-37*, GFP::RHO-aggregates were detected in oocytes, suggesting at least modestly effective depletion of gene expression during RNAi (Fig. 3a). However, GFP::MEX-3 protein appeared unchanged from a largely decondensed state, and quantitative analysis showed no significant increases in MEX-3 condensation (Fig. 3). In a minority of *vha-12(RNAi)* and *vha-2(RNAi)* worms (3 of 14 and 2 of 10, respectively), we detected a slight increase in the extent of MEX-3 condensation. Some of these oocytes also appeared more stacked, which may indicate a lack of sperm signal caused the MEX-3 condensates (Jud et al. 2008) (Fig. 1a). Alternatively, the low-penetrance phenotype may reflect a modest role for these 2 genes in preventing ectopic MEX-3 condensation or occur due to the limitations of RNAi efficacy. After RNAi of *spcs-1*, MEX-3 appeared largely decondensed, but we did not detect the expected phenotype of ectopic GFP::RHO-1 aggregates (Supplementary Fig. 2; Table 1) (Samaddar et al. 2021). Therefore, we cannot make any conclusions regarding the possible regulation of MEX-3 phase transitions by *spcs-1*. Taken together, the RHO-1 regulator genes required for V-ATPase assembly and docking, lysosomal acidification, regulation of mitochondrial membrane potential, and the ESCRT proteins do not appear to have large roles in regulating MEX-3 phase transitions in oocytes. Thus, we conclude that the genetic networks that prevent ectopic MEX-3 condensation and those that inhibit ectopic RHO-1 aggregates only partially overlap.

## Conclusions and considerations

High-quality oocytes are paramount to successful reproduction. Indeed, the ability of oocytes across species to maintain their quality during extended delays in fertilization is quite remarkable. Recent attention has focused on identifying mechanisms that protect oocytes from cytosolic damage such as protein misfolding and aggregation (Bohnert and Kenyon 2017; Samaddar et al. 2021; Harasimov et al. 2024; de Souza et al. 2025). At the same time, oocytes must protect maternal mRNAs and their partner RNA-binding proteins. The extent to which these processes are coordinated at a molecular level has not been examined. In this targeted genetic screen, we identified 6 novel regulators of MEX-3 phase transitions and obtained strong evidence that the regulatory network of protein aggregate clearance is distinct from the regulation of MEX-3 phase transitions in the oocyte.

One limitation in our study is that depletions that interfere with the oocyte maturation signal would also induce MEX-3 condensates in hermaphrodite oocytes (Jud et al. 2008). We cannot rule out the possibility that maturation is altered in the depletions. However, if this was the case, we should detect the characteristic stacking of oocytes in worms that lack a maturation signal (see arrested oocytes in Fig. 1a). We did not detect a stacked oocyte phenotype, consistent with prior studies (Fig. 2; Samaddar et al. 2021). In addition, partial depletion of several novel regulators results in embryonic lethality, which indicates oocyte maturation is not arrested (Poteryaev et al. 2005; DeBella et al. 2006; Bonner et al. 2013). This observation may be relevant since the method of RNAi by feeding often results in partial depletions.

Several newly identified MEX-3 regulators also modulate ER architecture (*copb-2*, *pbs-7*), consistent with prior studies linking these processes in oocytes (Langerak et al. 2019; Samaddar et al. 2021; Elaswad et al. 2024). Conversely, genes that appear to prevent RHO-1 aggregation but not MEX-3 condensation, *vha-12*, *vha-2*, and *atp-3*, do not affect ER morphology. Although we do not have evidence that MEX-3 diffuses laterally on the ER, these correlative findings are consistent with the possibility that expanded ER sheets promote MEX-3 condensation by restricting molecular diffusion, thereby lowering the concentration threshold for protein condensation (Snead and Gladfelter 2019; Elaswad et al. 2024).

Our identification of novel inhibitors of ectopic MEX-3 condensation advances our understanding of RNA-binding protein phase transitions in maturing oocytes. The results provide a foundation for future studies to probe the mechanisms by which these genes modulate MEX-3 phase and determine whether any genes have more global roles in regulating RNA-binding proteins phase transitions in maturing oocytes. Because aberrant phase transitions of hMex3 alter its interactions with P-bodies, modulate mRNA degradation, and are associated with the progression of colorectal cancer (Chen et al. 2024), our findings may have applications beyond oocyte quality and fertility.

## Supplementary Material

jkaf266_Supplementary_Data

## Data Availability

All *C. elegans* strains are available at the CGC and upon request. The authors state that all data necessary for confirming the conclusions are present within the article, figures, tables, including supplementary information.

## Supplemental material

Supplemental material available at G3 online.
