# Supplementary material for: Targeted RNAi screen reveals novel regulators of RNA-binding protein phase transitions in Caenorhabditis elegans oocytes
Source: G3 (Bethesda). 2025 Nov 10;16(1):jkaf266. doi: 10.1093/g3journal/jkaf266 (PMC12774588; doi:10.1093/g3journal/jkaf266)
Supplement: jkaf266_Supplementary_Data [file jkaf266_supplementary_data.zip › Supplementary_Figure_Legends_G3-2025-406328.docx]

**Supplementary Figure Legends**

**Supplementary Figure 1.** Levels of MEX-3 do not appear to increase in depletions causing ectopic condensation. The mean fluorescence intensity of MEX-3 in the -1 to -3 oocytes was determined using Fiji. Each dot represents the integrated density divided by the area of the ROI. Kruskal-Wallis test. *** P <0.001; **** P <0.0001. ns is not significant. n=6-11 worms.

**Supplementary Figure 2.** RNAi depletion of *spcs-1* did not reveal phenotypes for MEX-3 or RHO-1. Confocal micrographs of negative control, *lacZ(RNAi),* and *spcs-1(RNAi)* in GFP::MEX-3 and GFP::RHO-1 strains. No RHO-1 aggregates were detected, indicating the RNAi may not have been effective.
