# Supplementary figures and images for: Targeted RNAi screen reveals novel regulators of RNA-binding protein phase transitions in Caenorhabditis elegans oocytes
Source: G3 (Bethesda). 2025 Nov 10;16(1):jkaf266. doi: 10.1093/g3journal/jkaf266 (PMC12774588; doi:10.1093/g3journal/jkaf266)

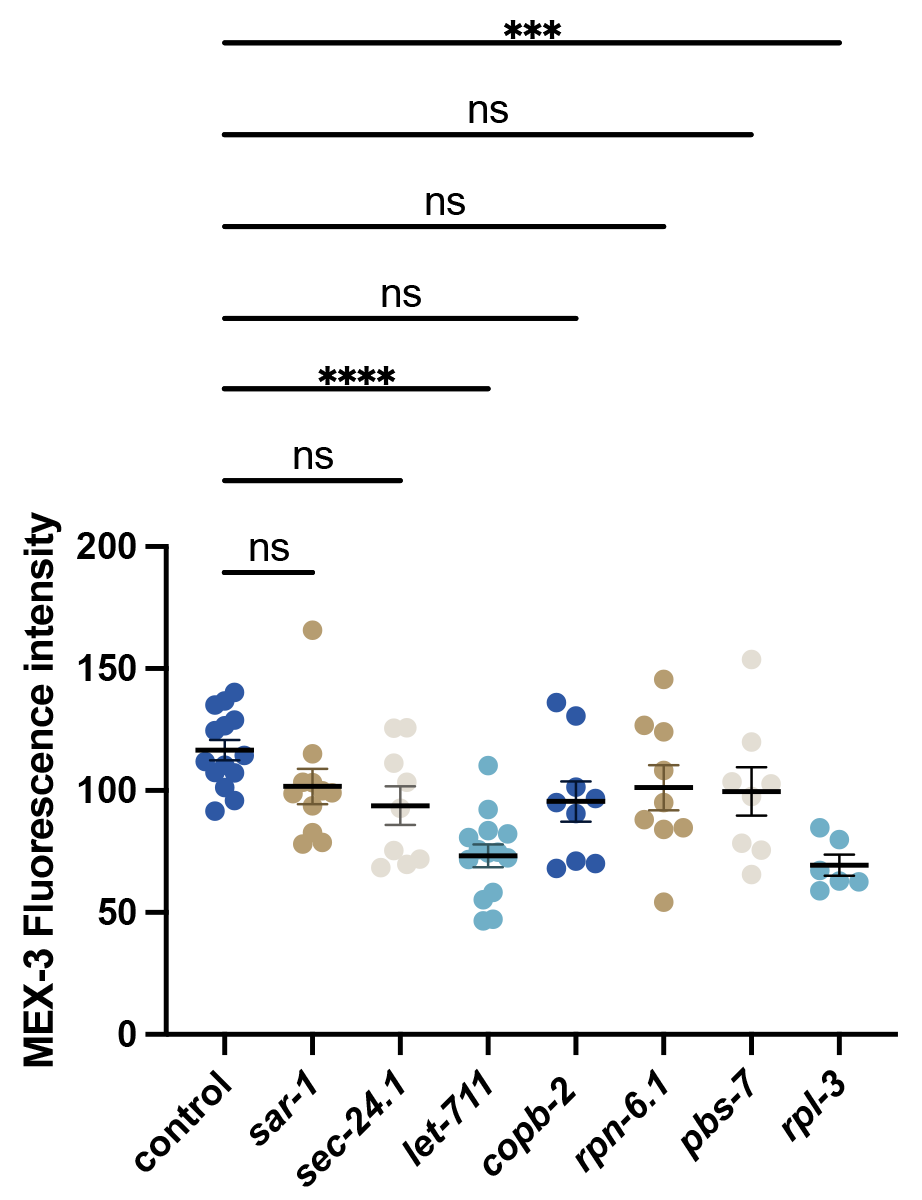

Supplement: jkaf266_Supplementary_Data [file jkaf266_supplementary_data.zip › Supplementary_Figure_1_G3-2025-406328.tif]

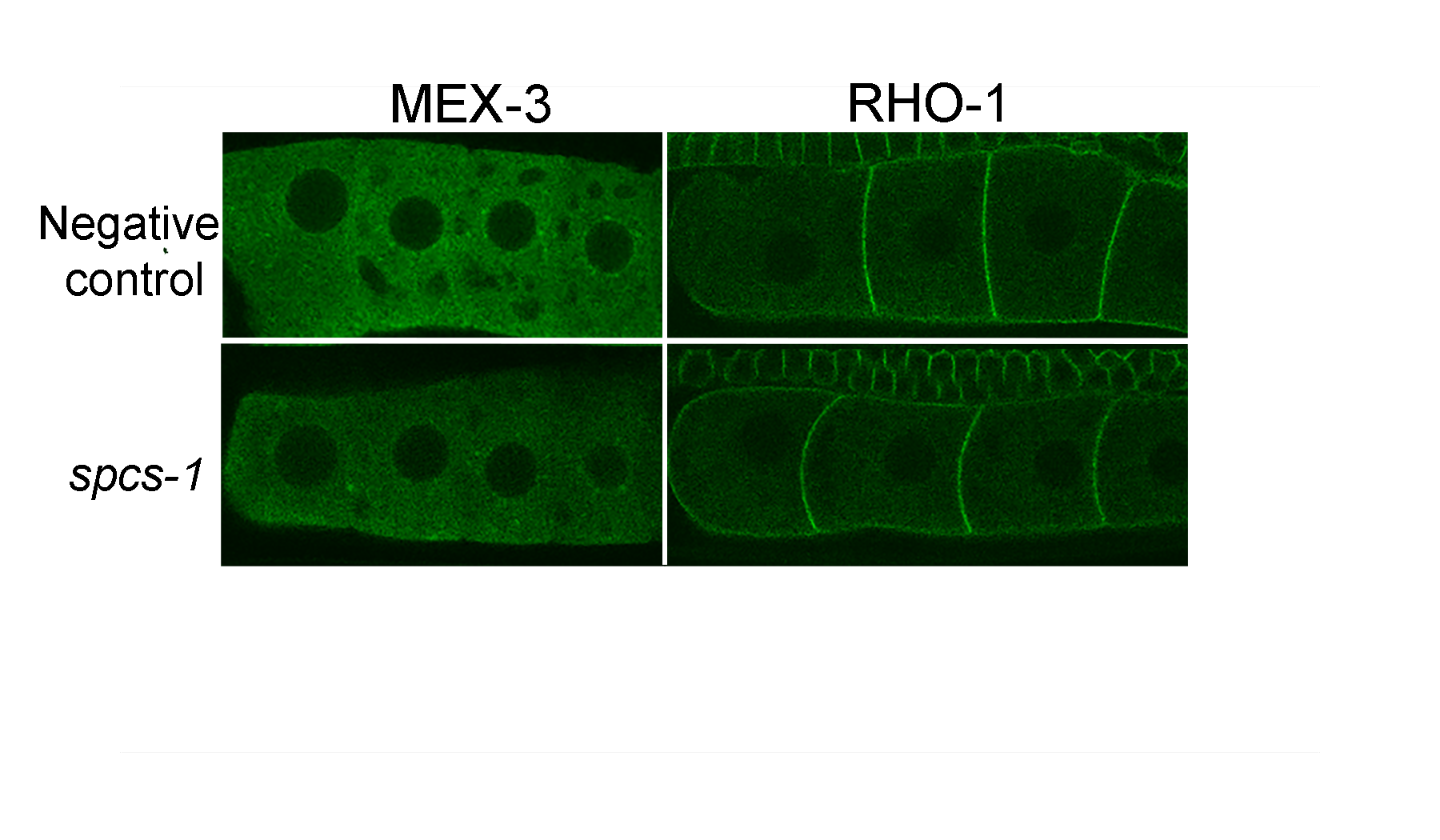

Supplement: jkaf266_Supplementary_Data [file jkaf266_supplementary_data.zip › Supplementary_Figure_2_G3-2025-406328.tif]
